# A longitudinal rat model for assessing postoperative recovery and bone healing following tibial osteotomy and plate fixation

**DOI:** 10.1186/s12891-023-06942-5

**Published:** 2023-10-31

**Authors:** Yingfang Fan, Charlotte P. Leape, Shannon Hugard, Madeline McCanne, Andrew Thomson, Gregory R. Wojtkiewicz, Michael J. Weaver, Jamie E. Collins, Mark Randolph, Ebru Oral

**Affiliations:** 1 Harris Orthopaedic Laboratory, Massachusetts General Hospital, Boston, MA USA; 2 Department of Orthopaedic Surgery, Harvard Medical School, 55 Fruit St. GRJ 1231, Boston, MA 02114 USA; 3 Boston College, Boston, MA USA; 4 Center for Systems Biology, Massachusetts General Hospital, Boston, MA USA; 5 Department of Orthopaedic Surgery, Brigham and Women’s Hospital, Boston, MA USA

**Keywords:** Trauma, Osteotomy, Fracture, Bone healing, Function, Gait analysis

## Abstract

**Background:**

Rodent models are commonly employed to validate preclinical disease models through the evaluation of postoperative behavior and allodynia. Our study investigates the dynamic interplay between pain and functional recovery in the context of traumatic osteotomy and surgical repair. Specifically, we established a rat model of tibial osteotomy, followed by internal fixation using a 5-hole Y-plate with 4 screws, to explore the hypothesis that histological bone healing is closely associated with functional recovery.

**Objective:**

Our primary objective was to assess the correlation between bone healing and functional outcomes in a rat model of tibial osteotomy and plate fixation.

**Methods:**

Seventeen male Sprague–Dawley rats underwent a metaphyseal transverse osteotomy of the proximal tibia, simulating a fracture-like injury. The resultant bone defect was meticulously repaired by realigning and stabilizing the bone surfaces with the Y-plate. To comprehensively assess recovery and healing, we performed quantitative and qualitative evaluations at 2, 4, 6, and 8 weeks post-surgery. Evaluation methods included micro-CT imaging, X-ray analysis, and histological examination to monitor bone defect healing. Concurrently, we employed video recording and gait analysis to evaluate functional recovery, encompassing parameters such as temporal symmetry, hindlimb duty factor imbalance, phase dispersion, and toe spread.

**Results:**

Our findings revealed complete healing of the bone defect at 8 weeks, as confirmed by micro-CT and histological assessments. Specifically, micro-CT data showed a decline in fracture volume over time, indicating progressive healing. Histological examination demonstrated the formation of new trabecular bone and the resolution of inflammation. Importantly, specific gait analysis parameters exhibited longitudinal changes consistent with bone healing. Hindlimb duty factor imbalance, hindlimb temporal symmetry, and phase dispersion correlated strongly with the healing process, emphasizing the direct link between bone healing and functional outcomes.

**Conclusions:**

The establishment of this tibia osteotomy model underscores the association between bone healing and functional outcomes, emphasizing the feasibility of monitoring postoperative recovery using endpoint measurements. Our overarching objective is to employ this model for assessing the local efficacy of drug delivery devices in ameliorating post-surgical pain and enhancing functional recovery.

**Supplementary Information:**

The online version contains supplementary material available at 10.1186/s12891-023-06942-5.

## Introduction

Long-bone fracture is one of the most common musculoskeletal injuries across all populations [1–3]. Tibia fractures, of which distal location of injury is the most common (37.8% [4]), have a high incidence of complications such as delayed union, nonunion [5, 6] and fracture-related infection [7]. The extent of injury and the difficulty in healing the tibia is due to multiple biological and biomechanical factors such as the involvement of soft tissue damage, the difficulty in wound coverage, the high incidence of periosteal damage and interruption of the intramedullary blood supply [8]. These fractures are associated with postoperative pain that can significantly affect the patients’ quality of life [9] and result in serious consequences such as loss of long-term income [10]. Data from clinical studies have shown that severe postoperative pain is associated with an increased risk of complications, a slow recovery process, delayed return to normal function, prolonged hospital stay, increased readmission rates, and higher total costs [11]. Thus, addressing pain associated with tibia fractures is significant. There is a need to improve the animal models relating to fracture with additional functional outcome measures to assess the efficacy of pain management. This can increase the translational value of preclinical studies which test different treatment tools and methods to address pain. While existing studies have focused extensively on bone healing, there is a need to explore the relationship between bone healing and functional recovery. Our study aims to address this need by investigating the use of the functional outcome measures of gait analysis and weight bearing in a rat tibia fracture model.

Small animal models present the advantage of studying long-bone fractures in a cost-effective and timely manner. We have chosen to use the rat due to the relative ease of placing implant materials locally and studying gait. The study of pain and pain-related behavior is most common in the rat in disease models of osteoarthritis; bone healing is not a focus in these models [12]. For modeling of long-bone metaphyseal fracture in the rat, the distal femur and the proximal tibia are the most widely used fracture sites. The proximity of the fibula to the tibia makes the creation of a tibial fracture/defect more difficult compared to that in the femur, requiring good surgical technique and stable plate fixation to avoid fibula damage. There are a variety of methods for the fixation of tibia fractures including K-wires, degradable rods, wire cerclage, T-shaped miniplate, and Y-shaped miniplate fixation [10]. Studies have shown high union rates and low incidences of infection, malunion, nonunion, and malalignment with plate fixation for the distal tibia fracture [13, 14]. We chose to use a standardized rat tibia osteotomy model to simulate a tibia fracture; using a Y-shaped miniplate for fracture fixation [10, 15] with a small modification of abutting the bone surfaces of the osteotomy instead of the 1-mm osteotomy gap used previously. We aimed to establish longitudinal assessment of pain and function in this long-bone fracture and healing model.

Most studies of long-bone fracture focus on bone healing [16–18] or fracture pain using allodynia [19]. Few animal models have applied postoperative function to study specifically postsurgical fracture pain and subsequent longitudinal recovery [20]. Our prior success in developing a rat model for periprosthetic joint infection incorporating gait analysis, and evaluation of weight-bearing symmetry, to gauge postoperative function in this orthopedic complication, has laid the groundwork for our current approach [21]. We hypothesize that the recovery of function following osteotomy and plate fixation can be assessed through gait analysis, with functional outcomes correlated to bone healing. In our investigation, we scrutinize the temporal progression and extent of bone healing through histology and micro-CT, respectively. Simultaneously, we analyze gait patterns, and weight-bearing asymmetry to comprehensively evaluate functional recovery and establish potential correlations between these parameters. Our overarching objective is to ultimately evaluate various local treatment regimens for pain management as part of a multifaceted strategy for enhancing pain control and functional recovery in the context of long-bone fractures.

## Materials and methods

### Animals and study timeline

This study was approved by the Institutional Care and Use Committee of Massachusetts General Hospital (2019N000031). The study is reported in accordance with ARRIVE guidelines. Seventeen adult male Sprague–Dawley rats were given facility chow and water ad libitum and randomly assigned (*n* = 3–5 per time point; 350-400 g; Charles River, Wilmington, MA). Diet supplementation was given a couple days after surgery to help rats recover their weight after an observed weight decline. The timeline of all procedures is shown in Figure S1. Animals were acclimatized for three days preoperatively. On the day before surgery, we performed preoperative baseline measurements. Postoperatively, video recording was performed for gait analysis as well as weight bearing measurements and serum α-2-Macroglobulin (α2M) ELISA analysis for inflammation. Euthanasia was carried out by administering an intraperitoneal injection of pentobarbital euthanasia solution (Euthasol®) at a dosage of 100 mg/kg on days 14, 28, 42, and 56 (with *n* = 3–5 for each timepoint), followed by the collection of bone samples. Micro-CT imaging and histology was performed ex vivo. One animal assigned to be euthanized at day 14 died prematurely due to causes unrelated to the trauma.

#### Surgical procedure: tibia osteotomy and plate fixation

**Fig. 1:**
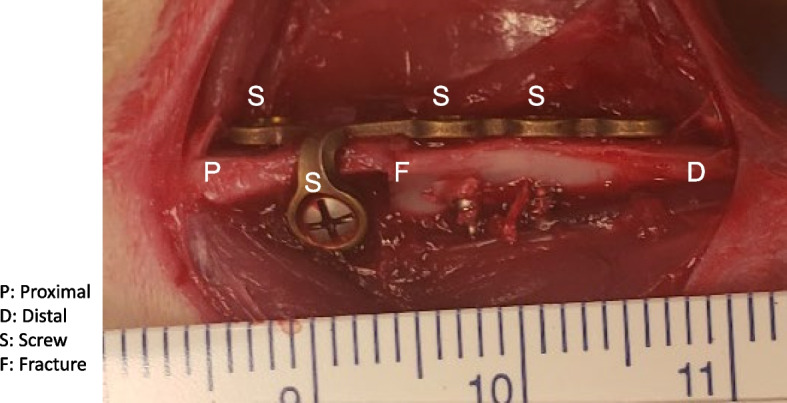
Photograph of tibia right after the surgery. Orientation from proximal (P) to distal (D). Fracture (F) was created and fixed with 5-hole Y-plate and screws (S)

Rats (body weight: 250–350 g; age 10–11 weeks) received preoperative buprenorphine (0.05 mg/kg, IP) 30 min before isoflurane anesthesia (1–3% isoflurane in 1L of O_2_/min for maintenance). Leg fur on the surgical limb (right leg) was shaved, and the skin was scrubbed with 10% povidone-iodine solution. A 3–4 cm skin incision was made, and the muscle was cleaned from the bone to expose the knee joint and the middle tibia of the right leg. During surgery, a metaphyseal transverse osteotomy of the proximal tibia was created by TPS Sagittal Saw (Stryker, MI, USA) 5 mm distal to the tibia plateau, and it was fixed by abutting the bone surfaces with a 2.3 cm long and 0.7 mm thick 5-hole Y-plate (Synthes, CO, USA), using four screws (Synthes, CO, USA) (Fig. 1). The wound was closed using 5–0 Vicryl deep dermal sutures, and the skin was closed using staples. All animals received postoperative buprenorphine (0.05 mg/Kg, IP) at 12-h intervals for 72 h.

### In-vivo endpoint measurements

#### Blood collection and analysis

The animals were weighed to ensure their overall health (Figure S2). Blood (~ 150 μL) collected from the lateral tail vein was centrifuged at 1500 g for 15 min. The serum was stored at -20 °C. The α2M ELISA (ab157730, Abcam) was performed to determine the concentration of α2M in serum. The concentration ratios were calculated using the baseline concentration for each rat. The number of measurements per timepoint are shown in Supplementary Table S1.

#### Static weight-bearing

Hindlimb weight-bearing protocol was guided by previous literature [22]. Subjects were placed in an acrylic enclosure over a pressure sensing mat (Walkway, TekScan, Inc.). Recording was conducted for 2 min to obtain a ten-second stationary window for all four paws. Videos were analyzed by taking the ratio of pressure units between right and left hind limbs. Pressure readings were averaged over the selected static weight-bearing window. The number of measurements per timepoint are shown in Supplementary Table S2.

#### Gait analysis

A walking arena was built for gait analysis as per guidance provided in the GAITOR Suite [23]. Gait videos were recorded at 400 fps and approximately 1350 microseconds of exposure time (PROMON U750, AOS Technologies, Switzerland). Data collection occurred on the pre-operative day and postoperative days 1, 3, 7, 14, 28, 42, and 56. The number of animals and the number of gait observations for each timepoint are shown in Supplementary Table S3. All gait trials occurring within 10 min of arena time were recorded for each individual. We maintain a database of healthy animals without injury to determine normal ranges (140 animals) and to calculate residuals for velocity- and weight-dependent metrics. Analyzed metrics included duty factor, temporal symmetry, spatial symmetry, and phase dispersion determined using toe-off (TO) and foot strike (FS) timepoints [21].

Gait videos were analyzed by ImageJ. Using the dorsal view, frames depicting flush contact of each paw with the glass floor were identified. Using the ventral view camera, 1–5 and 2–4 toe spread (5 measurements per animal) were measured manually. The ratio of these measurements between surgical (right) and control (left) paw was calculated. The number of observations for toe spread measurements are shown in supplementary Table S4.

#### X-ray

Lateral radiographs of all operative knees were obtained on the first day after the surgery (POD 1). The rats were anesthetized with isoflurane (1–3% isoflurane in 1L of O_2_/min) and placed on a high-resolution digital radiographic plate (Cuattro Hub, Heska, Loveland, CO). The output voltage was 54kVp and the exposure time was 0.075 s.

### Ex-vivo endpoint measurements

#### Micro-CT

The screws were removed from the tibias at sacrifice, which were imaged using X-ray and μ-CT (Siemens Inveon) with 720 projections (1600 ms exposure time/projection) with an 80 kV, 500 μA x-ray tube and reconstructed using a modified Feldkamp cone-beam reconstruction algorithm (COBRA, Exxim Inc., Pleasanton, CA) into a 512 × 512 × 800 matrix with 41.8-micron isotropic voxels. Parameters were measured on a 5 mm section and segmented using a combination of thresholding and manual segmentation (Amira) to determine total volume (TV), total bone volume (BV), bone volume fraction BV/TV, cortical and osseous callus volume (Ct.V and Cl. V, respectively), and osseous callus and cortical callus densities (Cl.BMD and Ct.BMD, respectively). Osseous callus fraction (Cl.V/total Cl.v, where Cl. V included the soft callus) was also calculated. For each sample in the 5 mm section, we manually drew a region of interest (ROI). One ROI was placed in the soft tissue (marrow), while another was placed at the center of the cortical bone to avoid partial volume effects. Soft tissue was designated as having Hounsfield units (HU) within 3 standard deviations of that of the soft tissue ROI. Bone was designated at least 3 standard deviations above the mean of the soft tissue ROI and cortical bone was designated to within 3 standard deviations below that of the mean of the cortical bone ROI. The callus was manually segmented with threshold separation between the soft and hard callus and osseous and cortical callous boundaries were determined in the manner described above. The fracture volume was manually drawn in only the soft tissue region (with the upper boundary value 3 standard deviations within the soft tissue and the lower boundary as the lowest value within the image since the fracture can just be air within the image). In this particular fracture there was a bone fragment in the middle of the fracture cavity that was unattached to either side of the cortex and that was included in the fracture volume. The soft tissue is also visible in the fracture cavity. The number of observations for bone healing parameter measurements are shown in Supplementary Table S5.

#### Histological analysis

The tibias were harvested and fixed in 4% formalin for 24-48 h, followed by decalcification in Versenate ethylenediaminetetraacetic acid (EDTA) decalcification solution (American MasterTech Scientific, Lodi, CA) at room temperature for 28 days. After histological processing, and embedding in paraffin, coronal Sects. (5 um) were cut through the fracture region and stained with hematoxylin and eosin (H & E) or Giemsa. Representative images were scanned and analyzed using high resolution image analysis software (NanoZoomer Digital Pathology, Meyer Instruments, Houston, TX). The staining was conducted for qualitative assessment, providing a broad understanding of cellular presence and distribution without quantitative cell counting.

### Statistical analyses

We followed the method of Kloefkorn et al*. *[24] to adjust stride length and step width for the animals’ velocity and weight. Data for each rat were averaged over each trial per day so that each animal contributed at most one observation per study day. We present descriptive statistics by study day and experimental group for each parameter of interest. We used paired t-test (parametric) and Wilcoxon Signed Rank test (non-parametric) to compare the change from pre-op on each study day. To assess the correlation between each study parameter (i.e., gait, toe spread) we used Pearson’s correlation coefficient with Fisher’s Z transformation to provide 95% confidence intervals. For the micro-CT analysis, we employed ANOVA with Dunnett's test for pairwise comparisons (comparing the week 6 and 8 timepoints to week 4), Welch's correction for unequal variances to determine the significance of the observed differences. To detect a between-group difference of approximately 1.25 standard deviations with 80% power and alpha = 0.05 requires a sample size of 12 animals per group. Four animals per group provides > 80% power to detect a between group difference of 2.5 standard deviations.

## Results

### Bone healing evaluation

The radiographs showed that all plates were appropriately fixed within the tibia. There were obvious fracture gaps until week 6, after which there were no fracture lines (Figure S3).

#### Cortical bone healing

**Fig. 2:**
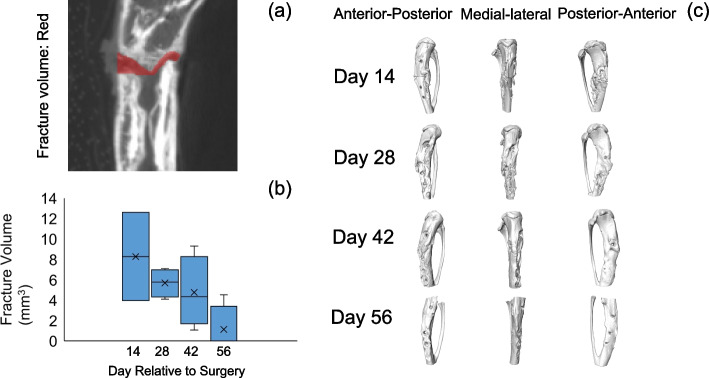
Quantitative analyses of tibia fracture volume at the osteotomy site. Designation of regions of interest for quantification overlaid on a micro-CT image cross-section (**a**); Fracture volume (**b**) and 3D representation of tibia with μCT (**c**). The fracture volume continued to decline through week 8. In this particular fracture, there was a bone fragment in the middle of the fracture cavity that was unattached to either side of the cortex and that was included in the fracture volume. The soft tissue is also visible in the fracture cavity

**Fig. 3:**
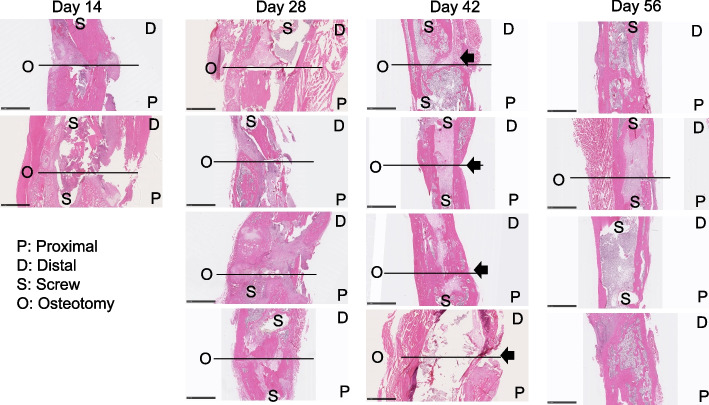
H&E stained coronal section of a tibia fracture. (scale bar = 2.5 mm) On the plated side of the images, cortical bone growth was less, and the neocortex was thinner compared to the other side (arrow)

Micro-CT was performed on the long bone column including the stabilizing soft tissue and callus. None of the samples, including the 2-week bone was unstable and showed any distinct movement. Designated ROI was overlaid on a μ-CT image cross-section for quantification (Figure S4). Total bone volume, total tissue volume, and bone volume fraction (BV/TV) did not differ between groups (Figure S5). Cortical bone volume increased with time, but its density did not change (Figure S6). Fracture volume declined through week 8 (Fig. 2a-c). We encountered limitation in obtaining data and making reasonable comparisons at week 2 and at week 8 due to no healing and significant healing, respectively. This led to a challenge in observing the fracture site and quantifying fracture volume and callus formation (continuity of the data values). Consequently, we had a large variation in the fracture volume at week 2 and did not have measurable data for the fracture and callus volumes at week 8 for most of the animals. Histologically, both the newly formed and existing cortical bone volume increased until week 8 (Fig. 3). On visual assessment of the images, it appeared that on the plated side, cortical bone growth was relatively less, and the neocortex appeared thinner compared to the other side at all timepoints except at week 8, where they seemed to be similar. It is important to note that these observations are qualitative in nature and based on visual assessment rather than quantitative measurements.

#### Intramedullary bone healing

**Fig. 4:**
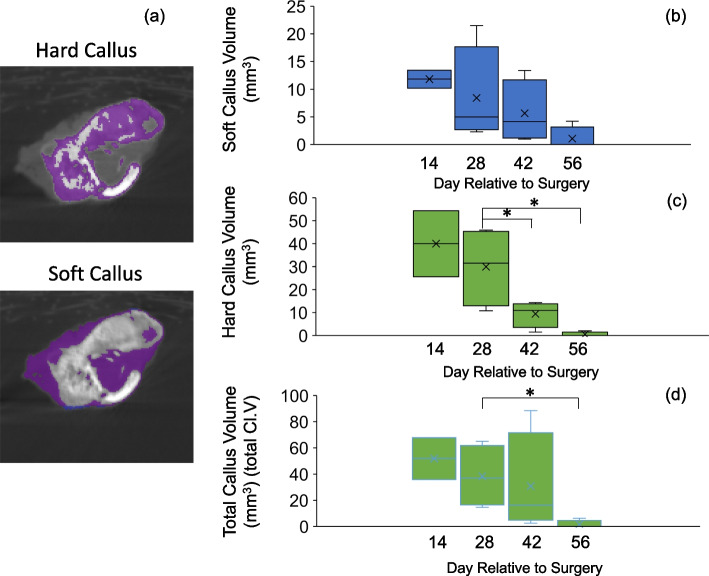
Quantitative analyses of tibia at the osteotomy site. Designation of regions of interest for quantification overlaid on a micro-CT image cross-section (**a**); total callus volume (**b**); soft callus volume (**c**) and hard callus volume (**d**). Asterisks indicate difference between groups (*p* < 0.05)

**Fig. 5:**
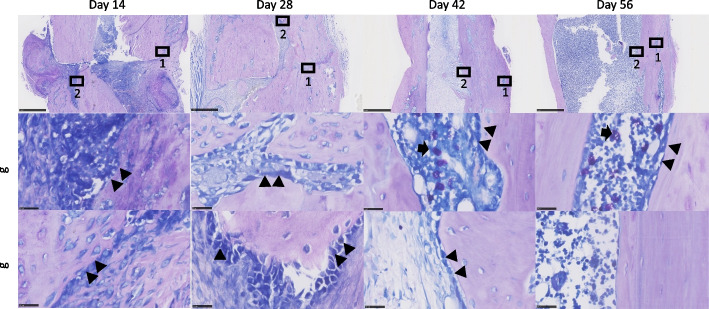
Giemsa staining of the fracture callus. (scale bar = 1 mm upper; scale bar = 25 um lower) Osteoblast (arrowhead) and osteoclast (arrow) are stained as blue. 1 represented fracture region; 2 represented bone integration during remolding. For samples with better healing and no discernible fracture line at day 42 and 56, we focused on any unhealed regions

**Fig. 6:**
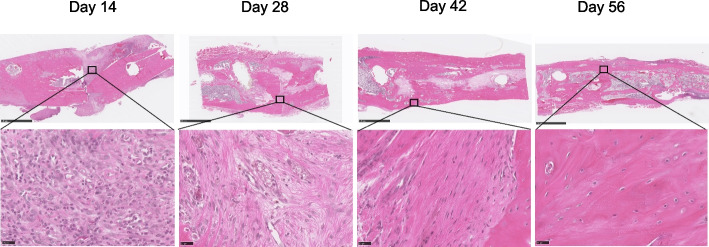
H&E staining of callus tissue. The hypercellular tissue turned to normal bone tissue after eight weeks. Scale bar = 2.5 mm, 25 μm

The volume of both the soft and hard calluses (Fig. 4a-c) was highest at 2 weeks, after which it decreased steadily until 8 weeks (Fig. 4d). Histologically, there was a larger amount of newly formed callus at week 2 and week 4 compared to that at week 6 and week 8. The thickness of the newly formed trabecular bone increased within the bone marrow canal until 8 weeks (Fig. 3). Giemsa staining highlights cellular dynamics in the tissue samples. Osteoblasts are stained blue in Giemsa staining and defined as cubically shaped cells of intermediate size residing on the bone surface (Fig. 5). There is plenty of osteoblasts around the defect surfaces at weeks 2 and 4. We also observed many osteoclasts in the unhealed cortical bone at weeks 6 and 8 (Fig. 5). There was no hypercellularity in the histological sections of bone at 8 weeks (Fig. 6).

### Inflammation analysis

**Fig. 7:**
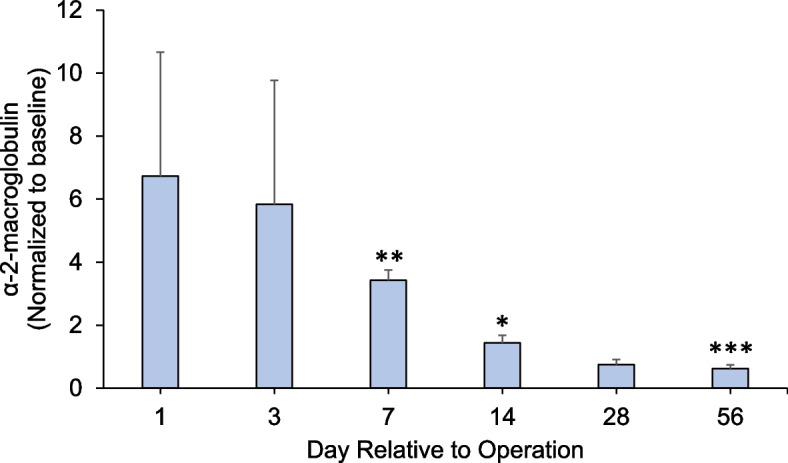
Post-sacrifice biomarker readings of alpha-2 macroglobulin (ng/mL) (*p < 0.05 vs. baseline). The inflammation marker alpha-2-macroglobulin decreased to the baseline by day 28

The concentrations of serum α2M increased initially after surgery and gradually decreased until 8 weeks with recovery to baseline at day 28 **(**Fig. 7). The α2M ratio was significantly lower than that at baseline on POD 7, 14, and 56 (*p < 0.05, **p < 0.01, ***p < 0.001; Fig. 7).

### Functional measurements

All rats showed partial weight bearing throughout the study. The ratio was significantly lower than that at baseline on POD 1, 3, 7, 14, and 28 (*p* < 0.05; Figure S7).

**Fig. 8:**
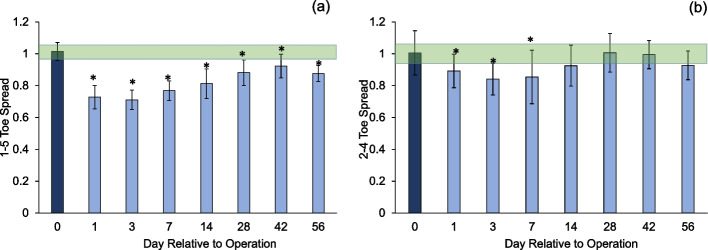
1–5 and 2–4 toe spread (*p < 0.05 vs. baseline). Shaded region indicates margin of error for database from healthy subject trials (1.01 ± 0.07 and 0.99 ± 0.11)

After surgery, both the 1–5 and 2–4 toe spread ratios were lower compared to that at baseline in all groups. The 1–5 toe spread was significantly lower than that at baseline at all time points (Fig. 8a), while the 2–4 toe spread had recovered to a level not significantly different from that at baseline by POD 28 (Fig. 8b).

**Fig. 9:**
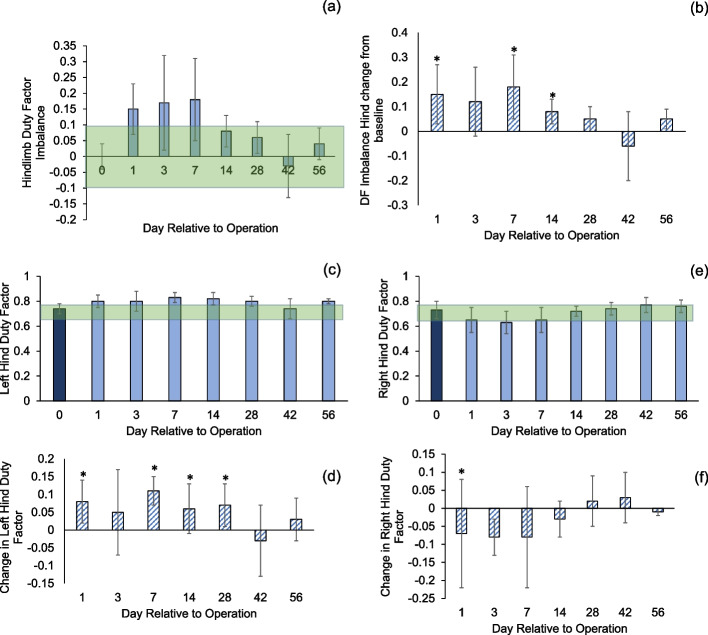
**a**, **b** Hindlimb duty factor imbalance presented as difference in duty factor percentage between left and right hindlimb (L-R). **c**, **d** Duty factor of the left (non-surgical) hindlimb. **e**, **f** Duty factor of the right (surgical) hindlimb. Asterisks indicate difference between groups (*p* < 0.05). Shaded area is the margin of error from database of healthy animals (0.00 ± 0.10, 0.71 ± 0.08, and 0.70 ± 0.07). Error bars are ± 1 SD

The animals showed a hindlimb duty factor imbalance outside the normal range until POD 28, and the animals showed an imbalance for the duration of the study (Fig. 9a and b). The differences between pre-op and post-op were significant for POD 1, 3, and 14 (Fig. 9b; *p* < 0.05). Simultaneously, the absolute value of the duty factor on the left (non-surgical side) was slightly elevated above the normal range (Fig. 9c). The difference between the groups was significant at POD 1, 7, 14, and 28 (Fig. 9d, *p* < 0.05). In contrast, right (surgical hindlimb) duty factor was below the normal range until POD 14 (Fig. 9e). The difference between the groups was significant at POD 1 (Fig. 9f, *p* < 0.05).

**Fig. 10:**
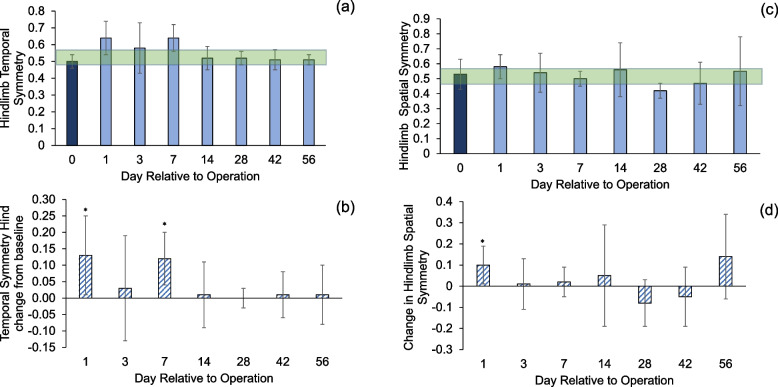
**a**, **b** Hindlimb temporal symmetry; values above 50% indicate delayed footstrike in right (surgical) limb. **c**, **d** Hindlimb spatial symmetry; values below 50% indicate right limb understepping. Asterisks indicate difference between groups (*p* < 0.05). Shaded area is the margin of error from database of healthy animals (0.50 ± 0.06 and 0.50 ± 0.08). Error bars are ± 1 SD

Hindlimb temporal symmetry of the animals increased after the surgery and recovered to the normal range during the study period (Fig. 10a). The difference from the baseline was statistically significant at POD 1 and 7 (Fig. 10b, *p* < 0.05). Hindlimb spatial symmetry increased right after surgery and then decreased below the normal range (Fig. 10c). The difference from the baseline was statistically significant at POD 1 (Fig. 10d, *p* < 0.05).

**Fig. 11:**
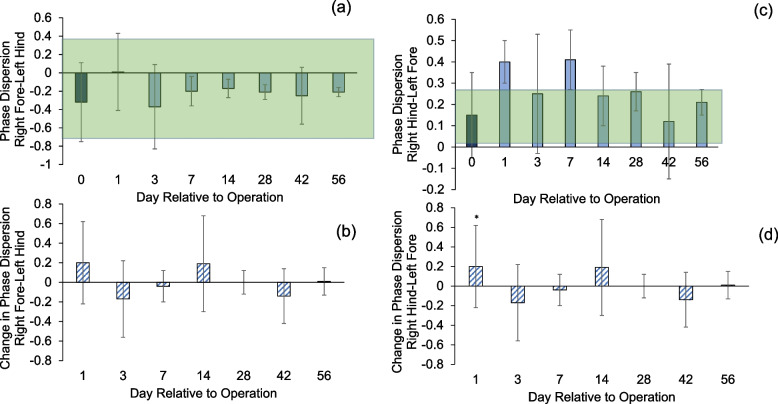
**a**, **b** RFLH (right fore-left hind) and (**c**, **d**) RHLF (right hind-left fore) diagonal phase dispersion. Asterisks indicate difference between groups (*p* < 0.05). Shaded area is the margin of error from database of healthy animals (-0.19 ± 0.64 and 0.15 ± 0.14). Error bars are ± 1 SD

The right fore-left hind (RFLH) phase dispersion was not different from the pre-operative values for all groups (Fig. 11a and b). Meanwhile, the right hind-left fore (RHLF) phase dispersion increased and was outside the normal range until POD 14 (Fig. 11c). The difference from the baseline was significant at POD 1 (Fig. 11d, *p* < 0.05).

**Fig. 12:**
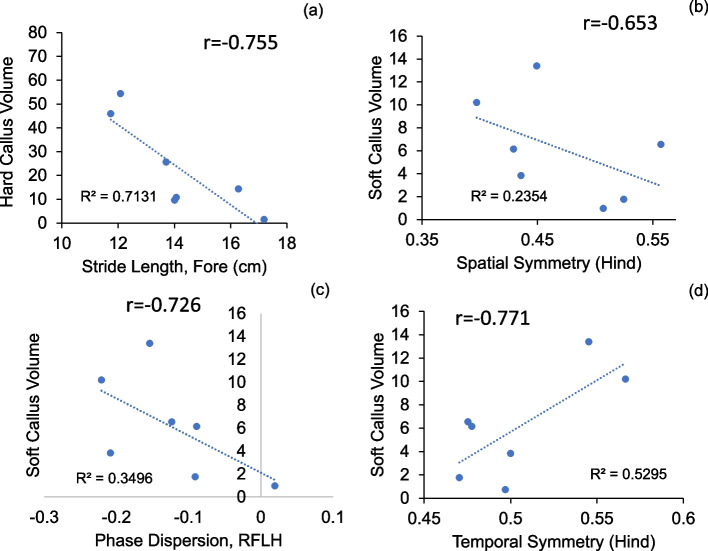
Correlations between bone healing and other parameters

**Fig. 13:**
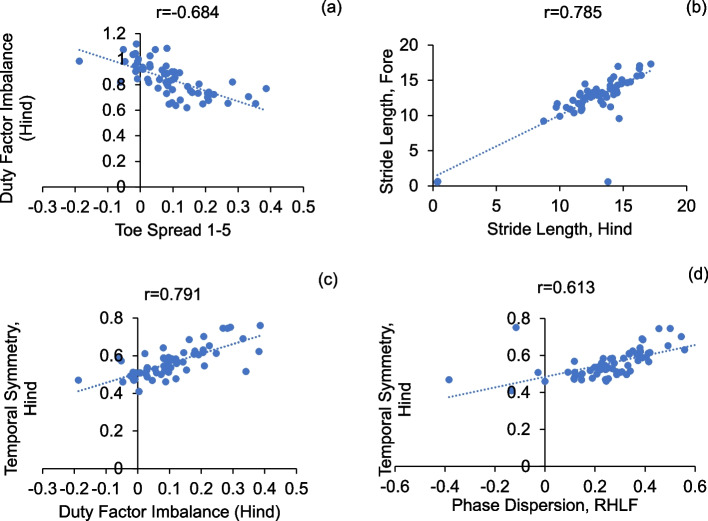
Correlations between functional parameters

**Fig. 14:**
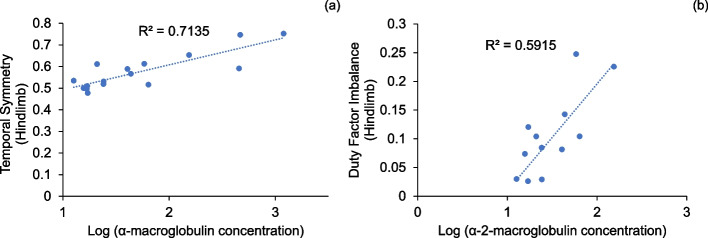
Correlations between inflammation and other parameters

We performed correlation analyses between bone healing parameters and other endpoint measurements, between functional parameters (gait parameters, toe spread and weight bearing), and between well-being parameters (toe spread, inflammation) (Table S6 and Figure S8). The plots of strongly correlated parameters (Pearson coefficient > 0.6) were plotted in Figs. 12, 13 and 14. Among bone healing parameters, hard callus volume had a negative correlation with stride length (Fig. 12a), soft callus volume had strong negative correlations with phase dispersion RHLF and hindlimb spatial symmetry and a strong positive correlation with hindlimb temporal symmetry (Fig. 12b-d). Among functional parameters, hindlimb duty factor imbalance showed a strong negative correlation with 1–5 toe spread (Fig. 13a), the forelimb stride length and the hindlimb stride length showed a strong positive correlation (Fig. 13b) and temporal symmetry of the hindlimbs was positively correlated with duty factor imbalance and right hind-left fore (RHLF) phase dispersion (Fig. 13c, d). Finally, systemic inflammation had strong correlations with hindlimb temporal symmetry (Fig. 14a) and hindlimb duty factor imbalance (Fig. 14b).

## Discussion

Plate fixation can be a stable and reproducible tool to perform reliable union [25]. We used Y-shape plate fixation (Fig. 1), which is widely practiced clinically. The healing of bone defects, which is slow [18], results from membranous bone produced by the mesenchymal stem cells of the trabecular bone marrow. In our study, declining fracture volume (Fig. 2) with variable comprehensive healing at six weeks (Figs. S3 and 3) indicated that union was achieved but healing continued until 8 weeks. Cortical bone growth in the tibia was slower and the neocortex was thinner on the plate side (Fig. 3), indicating that load-sharing by the plate limited callus formation surrounding the intramedullary defect. These results are consistent with the literature, where the amount of callus has been shown to be inversely proportional to the extent of immobilization [26]. Despite this delay in bone healing, plate fixation resulted in union for all animals.

Endochondral bone formation during fracture healing comprises the early inflammatory, repair and remodeling stages. Mechanical injury leads to an acute inflammatory response followed by osteoclast-mediated resorption [27]. Generally, α2-macroglobulin (α2M) acts as a protease inhibitor and negatively regulates coagulation by suppressing the activation of thrombin. α2M reportedly behaves as a carrier protein that binds with some cytokines or growth factors to harmonize the immune system. We have chosen to use it because it is a good analogue in rats for acute systemic inflammation (especially in infection) [21], akin to C-reactive protein (CRP) in humans. Previous research has indicated its role as an acute-phase reactant and a broad marker of systemic inflammation [28, 29]. Moreover, in the context of fracture healing, serum α-2-macroglobulin’s potential to reflect the systemic inflammatory milieu may hold relevance beyond its direct association with fracture repair. Systemic inflammation was significantly increased at 2 weeks and recovered to baseline after 4 weeks, suggesting that the acute early inflammatory phase of bone defect healing was complete by this time. This is consistent with histological results (Fig. 6), which showed no inflammatory cell infiltration and more bone tissue formation after 4 weeks. Normal recruitment of inflammatory cells, which is critical in the bone healing response without complications, was observed. A conversion of the preliminary soft callus to a hard callus was in progress at 2 weeks with a medullary callus supporting the bridging soft callus (Fig. 4). There was less cortical callus volume around 4 weeks, while its density also increased after 4 weeks (Fig. 4b), suggesting that repair and remodeling started at around 4 weeks and continued throughout the study. Clinically, bony callus formation has been shown to occur between days 11 to 28 [30]. Osteoblasts directly generated from mesenchymal stem cells play a crucial role in the maintenance and regeneration of bone mass in fully stabilized defects. Figure 5 shows the osteoblast cells along the bone surfaces adjacent to the defect surfaces at weeks 2 and 4, consistent with the clinical scenario. Remodeling the bony callus is traditionally considered the last stage of bone fracture repair [31]. Bone degradation by osteoclasts, which is an essential component of callus remodeling [31, 32] starts around day 18 and can last months to years. We found many osteoclasts on the unhealed cortical bone until week 8 as indicators of continued remodeling.

The systemic inflammatory markers, weight-bearing asymmetry analysis, and toe-spread present a comprehensive evaluation of the animals’ wellbeing. All rats lost weight after the surgery, a reasonable outcome for acute and systemic inflammation associated with the trauma and surgery (Figure S2). Although weight bearing was asymmetric throughout the study (Figure S7), the rats exhibited near-normal weight bearing close to the healthy range after 4 weeks. Full weight bearing has been associated with critical ossification of the osteotomy gap [33], supporting the direct bone healing data discussed above and suggesting that the critical ossification of this defect was observed around 4 weeks. Toe-spread (Fig. 8) was established as a good indicator of the animals’ recovery in our previous study on periprosthetic infection [21]. We confirmed its use in the present model of traumatic injury and recovery after surgical stabilization.

Our study investigated to what extent gait outcomes correlated with fracture healing progression. We have identified hindlimb duty factor imbalance, hindlimb temporal symmetry, and the right (surgical) hindlimb to left forelimb (RHLF) phase dispersion to be the best indicators of the function of the animals (Figs. 9, 10 and 11). Gait changes observed during the healing process may be influenced, in part, by critical ossification; there was a lot of variation in gait and weight bearing before 2 weeks and most significant imbalances recovered close to healthy ranges at 4 weeks (Figs. 9, 10 and 11). Based on the state of the bone healing observed via micro-CT and histology (Fig. 3), hard callus volume greatly influenced load bearing, the association of which has been suggested [17]. In terms of the correlation of bone healing parameters to gait, weight bearing parameters, reflex response and inflammation, hard and especially soft callus volume both correlated well with temporal and spatial parameters of gait (Fig. 12), with higher callus volume causing a larger variation from normal function. The strong correlation of duty factor imbalance with 1–5 toe spread (Fig. 13a) confirms the use of toe spread as a versatile tool for assessing well-being and also suggested that it can be a functional parameter. In addition, similar to our previous work, the gait parameters; temporal symmetry of the hindlimb, duty factor imbalance and phase dispersion correlations (Fig. 13), supported their use as appropriate gait parameters in this model to evaluate functional recovery.

The fact that the 1–5 toe spread did not recover to normal range in 8 weeks despite (1) both showing positive correlations with duty factor imbalance, (2) the values of 1–5 toe spread at 8 weeks being lower than that at 6 weeks and (3) that the spatial symmetry staying lower than the normal range at 8 weeks. This results suggested incomplete and continuing recovery in the soft tissues surrounding the injury. The strong effect of inflammation on the time-dependent gait parameters (Fig. 14a and b) showed that functional deficits in the first 2 weeks (Figs. 9 and 10), where inflammation was high (Fig. 8), were largely influenced by it. These results support the complementary use of these parameters in understanding the recovery timeline with respect to bony and soft tissue healing. In addition, combined with our earlier work on periprosthetic infection, these results clarify the relative importance of each endpoint measurements in the tibia fracture model (via osteotomy) compared to joint replacement.

Given the study design of sacrificing animals at different days, the statistical power for detecting within animal changes is greater for the earlier timepoints (with more animals) compared to later timepoints that had fewer animals (e.g., 5 animals at day 56); the micro-CT analyses were conducted on 15 animals. Even with 4 animals, the assessment of longitudinal changes were sufficiently powered (to detect 2 standard deviation differences). However, we do note that we did not adjust for multiple testing. We do not have a treatment group in this study; the number of animals required for the comparison of treatments would also depend on the treatment effects. Although this is a long-term consideration and requires a pilot study, our data from our periprosthetic infection model using gait suggests that 6–8 animals would be sufficient for comparing treatments at different time points.

The present study has limitations. The clinical treatment of tibia fractures in the trabecular bone is more likely to be repaired by plate fixation. We could not differentiate this type of treatment in the rat. Besides, humans are not allowed to bear weight after fracture fixation for several weeks to hinder any significant migration of bone surfaces and we did not remove immobilization during the study; thus, the timeline of functional recovery would differ in the human. Finally, the rat’s quadrupedal biomechanics are not representative, and gait analysis is not meant to be directly translated to humans.

No animal models have applied postoperative function (gait) to study fracture pain and subsequent longitudinal recovery. The rat model developed at the AO institute [34, 35] has several factors that are differentiated from the current model; it is a femur fracture model, it involves a segmental defect aiming to simulate non-union, and it focuses on determining the trajectory of bone healing using radiographic, histological, and biomechanical techniques, but not function (gait). We are fundamentally interested in the tibia because of our long-term goal of studying tibia-specific complications. The long-term goal of developing this model is to compare the efficacy of locally administered non-opioid analgesics to current pain management methods in addressing post-surgical function/pain in a tibia osteotomy while preserving bone healing.

## Conclusions

In this study, we developed a rat tibia fracture model stabilized with plate fixation and conducted assessments of bone healing and functional recovery. Our findings indicate a progressive bone healing process correlated to functional parameters. The model’s direct translational implications to human tibia fractures are limited given inherent anatomical differences.

### Supplementary Information

**Additional file 1.** 

**Additional file 2. ** 

**Additional file 3.** 

**Additional file 4.** 

## Data Availability

The datasets used and/or analyzed during the current study are available from the corresponding author on reasonable request.

## References

[1] Heckman JD, Sarasohn-Kahn J (1997). The economics of treating tibia fractures. Bull Hosp Joint Dis.

[2] Puno RM, Teynor JT, Nagano J, Gustilo RB (1986). Critical analysis of results of treatment of 201 tibial shaft fractures. Clin Orthop Relat Res.

[3] Whittle A, Russell T, Taylor J, Lavelle D (1992). Treatment of open fractures of the tibial shaft with the use of interlocking nailing without reaming. J Bone Joint Surg Am.

[4] Iqbal HJ, Pidikiti P (2013). Treatment of distal tibia metaphyseal fractures; plating versus intramedullary nailing: a systematic review of recent evidence. Foot Ankle Surg.

[5] Boyd H, Anderson L, Johnston D (1965). Changing Concepts in the Treatment of Nonunion. Clin Orthop Relat Res (1976-2007).

[6] Darder A, Gomar F (1975). A series of tibial fractures treated conservatively. Injury.

[7] Foster AL, Moriarty TF, Zalavras C, Morgenstern M, Jaiprakash A, Crawford R (2021). The influence of biomechanical stability on bone healing and fracture-related infection: the legacy of Stephan Perren. Injury.

[8] Rommens PM, Coosemans W, Broos PL (1989). The difficult healing of segmental fractures of the tibial shaft. Arch Orthop Trauma Surg.

[9] Zhang L, Terrando N, Xu ZZ, Bang S, Jordt SE, Maixner W (2018). Distinct analgesic actions of DHA and DHA-derived specialized pro-resolving mediators on post-operative pain after bone fracture in mice. Front Pharmacol.

[10] Haffner-Luntzer M, Ignatius A (2020). Animal models for studying metaphyseal bone fracture healing. Eur Cell Mater.

[11] Barrington JW, Olugbode O, Lovald S, Ong K, Watson H, Emerson RH (2015). Liposomal bupivacaine: a comparative study of more than 1000 total joint arthroplasty cases. Orthop Clin North Am.

[12] Hammell DC, Zhang LP, Ma F, Abshire SM, McIlwrath SL, Stinchcomb AL (2016). Transdermal cannabidiol reduces inflammation and pain-related behaviours in a rat model of arthritis. Eur J Pain.

[13] Richard RD, Kubiak E, Horwitz DS (2014). Techniques for the surgical treatment of distal tibia fractures. Orthop Clin North Am.

[14] Zelle BA, Bhandari M, Espiritu M, Koval KJ, Zlowodzki M, Evidence-Based Orthopaedic Trauma Working G (2006). Treatment of distal tibia fractures without articular involvement: a systematic review of 1125 fractures. J Orthop Trauma.

[15] Mackert GA, Schulte M, Hirche C, Kotsougiani D, Vogelpohl J, Hoener B (2017). Low-energy extracorporeal shockwave therapy (ESWT) improves metaphyseal fracture healing in an osteoporotic rat model. PLoS One.

[16] Grewal BS, Keller B, Weinhold P, Dahners LE (2014). Evaluating effects of deferoxamine in a rat tibia critical bone defect model. J Orthop.

[17] Horstmann PF, Raina DB, Isaksson H, Hettwer W, Lidgren L, Petersen MM (2017). (*) Composite biomaterial as a carrier for bone-active substances for metaphyseal tibial bone defect reconstruction in rats. Tissue Eng Part A.

[18] Liu J, Li X, Zhang D, Jiao J, Wu L, Hao F (2018). Acceleration of bone defect healing and regeneration by low-intensity ultrasound radiation force in a rat tibial model. Ultrasound Med Biol.

[19] Freeman KT, Koewler NJ, Jimenez-Andrade JM, Buus RJ, Herrera MB, Martin CD (2008). A fracture pain model in the rat: adaptation of a closed femur fracture model to study skeletal pain. Anesthesiology.

[20] Minville V, Laffosse J-M, Fourcade O, Girolami J-P, Tack I (2008). Mouse model of fracture pain. Anesthesiology.

[21] Fan Y, Xiao Y, Sabuhi WA, Leape CP, Gil D, Grindy S (2020). Longitudinal model of periprosthetic joint infection in the rat. J Orthop Res.

[22] Schuelert N, McDougall JJ (2012). Involvement of Nav 1.8 sodium ion channels in the transduction of mechanical pain in a rodent model of osteoarthritis. Arthritis Res Ther.

[23] Jacobs BY, Lakes EH, Reiter AJ, Lake SP, Ham TR, Leipzig ND (2018). The open source GAITOR suite for rodent gait analysis. Sci Rep.

[24] Kloefkorn HE, Jacobs BY, Loye AM, Allen KD (2015). Spatiotemporal gait compensations following medial collateral ligament and medial meniscus injury in the rat: correlating gait patterns to joint damage. Arthritis Res Ther.

[25] Yoon RS, Bible J, Marcus MS, Donegan DJ, Bergmann KA, Siebler JC (2015). Outcomes following combined intramedullary nail and plate fixation for complex tibia fractures: a multi-centre study. Injury.

[26] Wraighte PJ, Scammell BE (2006). Principles of fracture healing. Surg Infect (Larchmt).

[27] Yu MD, Su BH, Zhang XX (2018). Morphologic and molecular alteration during tibia fracture healing in rat. Eur Rev Med Pharmacol Sci.

[28] Vandooren J, Itoh Y (2021). Alpha-2-macroglobulin in inflammation immunity and infections. Front Immunol.

[29] Zhao R, Wei X, Zhang C, Wu H, Xiang C, Li H (2022). alpha2-macroglobulin-rich serum as a master inhibitor of inflammatory factors attenuates cartilage degeneration in a mini pig model of osteoarthritis induced by "idealized" anterior cruciate ligament reconstruction. Front Pharmacol.

[30] Sheen JR, Garla VV. Fracture healing overview. StatPearls: StatPearls Publishing; 2021. 31869142

[31] Bahney CS, Zondervan RL, Allison P, Theologis A, Ashley JW, Ahn J (2019). Cellular biology of fracture healing. J Orthop Res.

[32] Yang X, Ricciardi BF, Hernandez-Soria A, Shi Y, Pleshko Camacho N, Bostrom MP (2007). Callus mineralization and maturation are delayed during fracture healing in interleukin-6 knockout mice. Bone.

[33] Nemecek E, Chiari C, Valentinitsch A, Kainberger F, Hobusch G, Kolb A (2019). Analysis and quantification of bone healing after open wedge high tibial osteotomy. Wien Klin Wochenschr.

[34] Gruber HE, Gettys FK, Montijo HE, Starman JS, Bayoumi E, Nelson KJ (2013). Genomewide molecular and biologic characterization of biomembrane formation adjacent to a methacrylate spacer in the rat femoral segmental defect model. J Orthop Trauma.

[35] Liu F, Wells JW, Porter RM, Glatt V, Shen Z, Schinhan M (2016). Interaction between living bone particles and rhBMP-2 in large segmental defect healing in the rat femur. J Orthop Res.

