# Supplementary material for: A longitudinal rat model for assessing postoperative recovery and bone healing following tibial osteotomy and plate fixation
Source: BMC Musculoskelet Disord. 2023 Oct 31;24:854. doi: 10.1186/s12891-023-06942-5 (PMC10617055; doi:10.1186/s12891-023-06942-5)
Supplement: Supplementary file 1 — Additional file 1. [file 12891_2023_6942_MOESM1_ESM.pptx]

## Slide 1
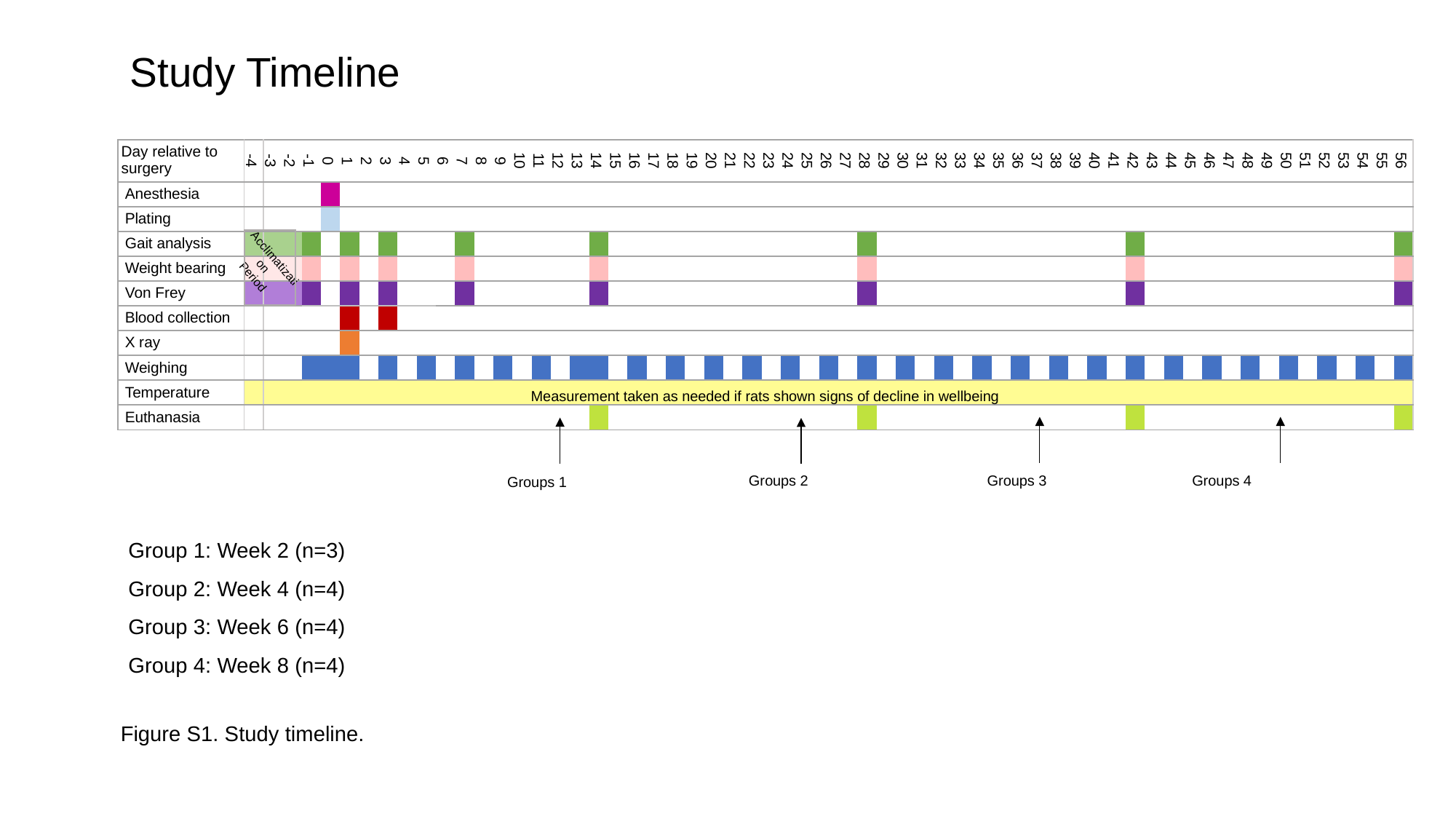

Study Timeline
| Day relative to surgery | -4 | -3 | -2 | -1 | 0 | 1 | 2 | 3 | 4 | 5 | 6 | 7 | 8 | 9 | 10 | 11 | 12 | 13 | 14 | 15 | 16 | 17 | 18 | 19 | 20 | 21 | 22 | 23 | 24 | 25 | 26 | 27 | 28 | 29 | 30 | 31 | 32 | 33 | 34 | 35 | 36 | 37 | 38 | 39 | 40 | 41 | 42 | 43 | 44 | 45 | 46 | 47 | 48 | 49 | 50 | 51 | 52 | 53 | 54 | 55 | 56 |
| --- | --- | --- | --- | --- | --- | --- | --- | --- | --- | --- | --- | --- | --- | --- | --- | --- | --- | --- | --- | --- | --- | --- | --- | --- | --- | --- | --- | --- | --- | --- | --- | --- | --- | --- | --- | --- | --- | --- | --- | --- | --- | --- | --- | --- | --- | --- | --- | --- | --- | --- | --- | --- | --- | --- | --- | --- | --- | --- | --- | --- | --- |
| Anesthesia | | | | | | | | | | | | | | | | | | | | | | | | | | | | | | | | | | | | | | | | | | | | | | | | | | | | | | | | | | | | | |
| Plating | | | | | | | | | | | | | | | | | | | | | | | | | | | | | | | | | | | | | | | | | | | | | | | | | | | | | | | | | | | | | |
| Gait analysis | | | | | | | | | | | | | | | | | | | | | | | | | | | | | | | | | | | | | | | | | | | | | | | | | | | | | | | | | | | | | |
| Weight bearing | | | | | | | | | | | | | | | | | | | | | | | | | | | | | | | | | | | | | | | | | | | | | | | | | | | | | | | | | | | | | |
| Von Frey | | | | | | | | | | | | | | | | | | | | | | | | | | | | | | | | | | | | | | | | | | | | | | | | | | | | | | | | | | | | | |
| Blood collection | | | | | | | | | | | | | | | | | | | | | | | | | | | | | | | | | | | | | | | | | | | | | | | | | | | | | | | | | | | | | |
| X ray | | | | | | | | | | | | | | | | | | | | | | | | | | | | | | | | | | | | | | | | | | | | | | | | | | | | | | | | | | | | | |
| Weighing | | | | | | | | | | | | | | | | | | | | | | | | | | | | | | | | | | | | | | | | | | | | | | | | | | | | | | | | | | | | | |
| Temperature | | | | | | | | | | | | | | | | | | | | | | | | | | | | | | | | | | | | | | | | | | | | | | | | | | | | | | | | | | | | | |
| Euthanasia | | | | | | | | | | | | | | | | | | | | | | | | | | | | | | | | | | | | | | | | | | | | | | | | | | | | | | | | | | | | | |
Acclimatization
Period
Measurement taken as needed if rats shown signs of decline in wellbeing
Groups 4
Groups 3
Groups 2
Groups 1
Group 1: Week 2 (n=3)
Group 2: Week 4 (n=4)
Group 3: Week 6 (n=4)
Group 4: Week 8 (n=4)
Figure S1. Study timeline.

## Slide 2
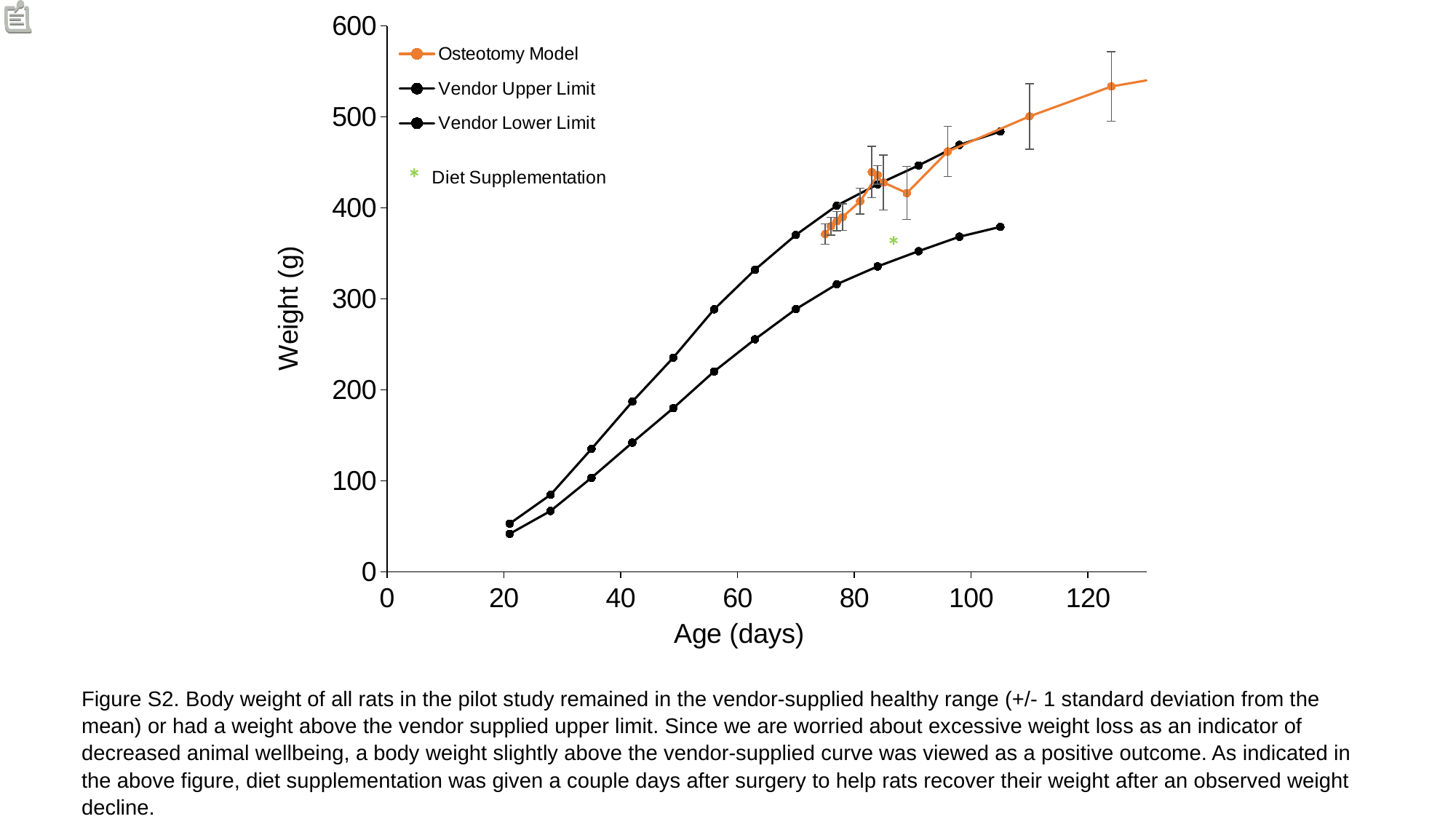

### Chart
| Category | Osteotomy Model | | |
|---|---|---|---|*
Figure S2. Body weight of all rats in the pilot study remained in the vendor-supplied healthy range (+/- 1 standard deviation from the mean) or had a weight above the vendor supplied upper limit. Since we are worried about excessive weight loss as an indicator of decreased animal wellbeing, a body weight slightly above the vendor-supplied curve was viewed as a positive outcome. As indicated in the above figure, diet supplementation was given a couple days after surgery to help rats recover their weight after an observed weight decline.
82
88

## Slide 3
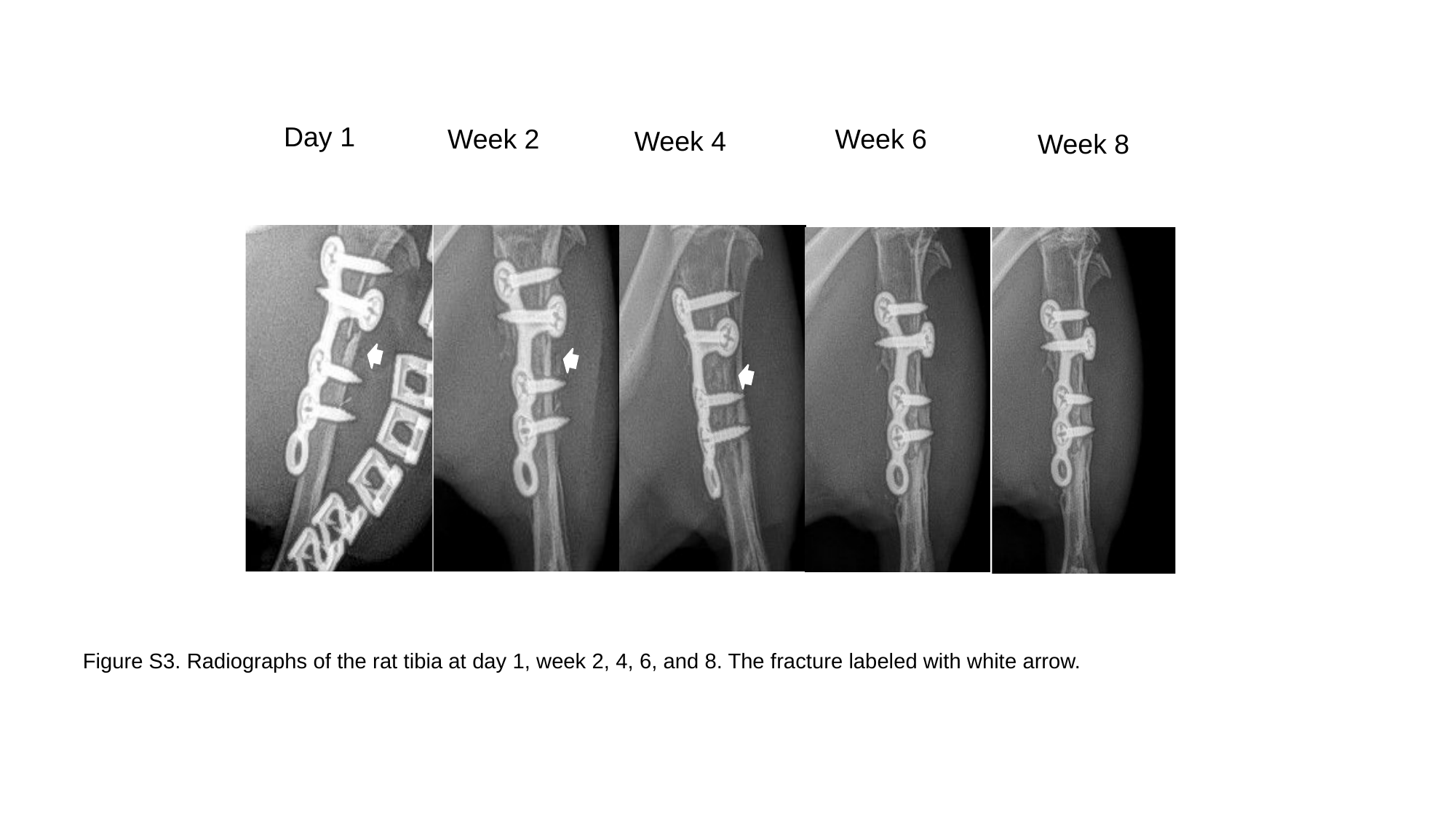

Day 1
Week 2
Week 6
Week 4
Week 8
Figure S3. Radiographs of the rat tibia at day 1, week 2, 4, 6, and 8. The fracture labeled with white arrow.

## Slide 4
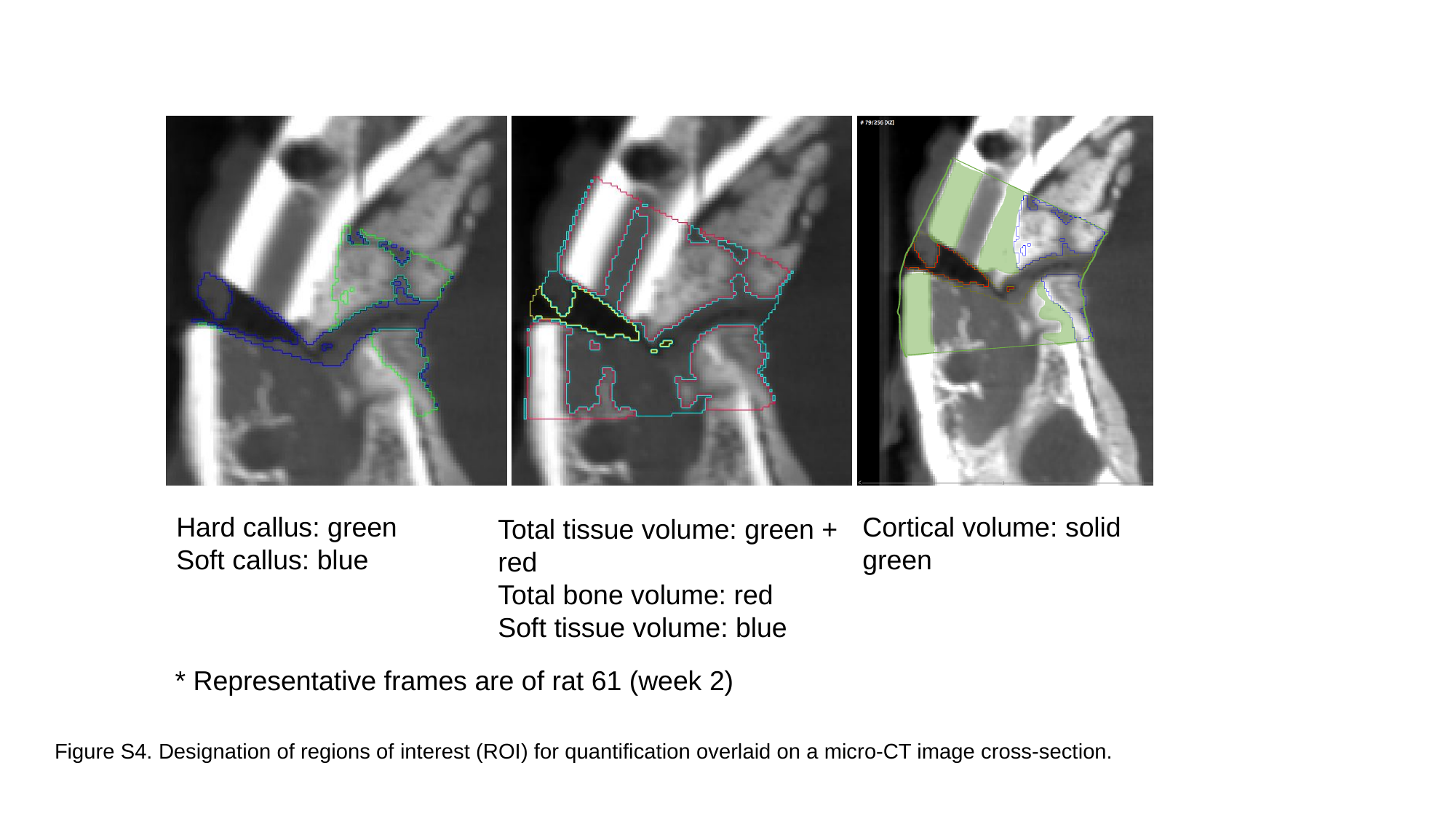

Hard callus: green
Soft callus: blue
Cortical volume: solid green
Total tissue volume: green + red
Total bone volume: red
Soft tissue volume: blue
* Representative frames are of rat 61 (week 2)
Figure S4. Designation of regions of interest (ROI) for quantification overlaid on a micro-CT image cross-section.

## Slide 5
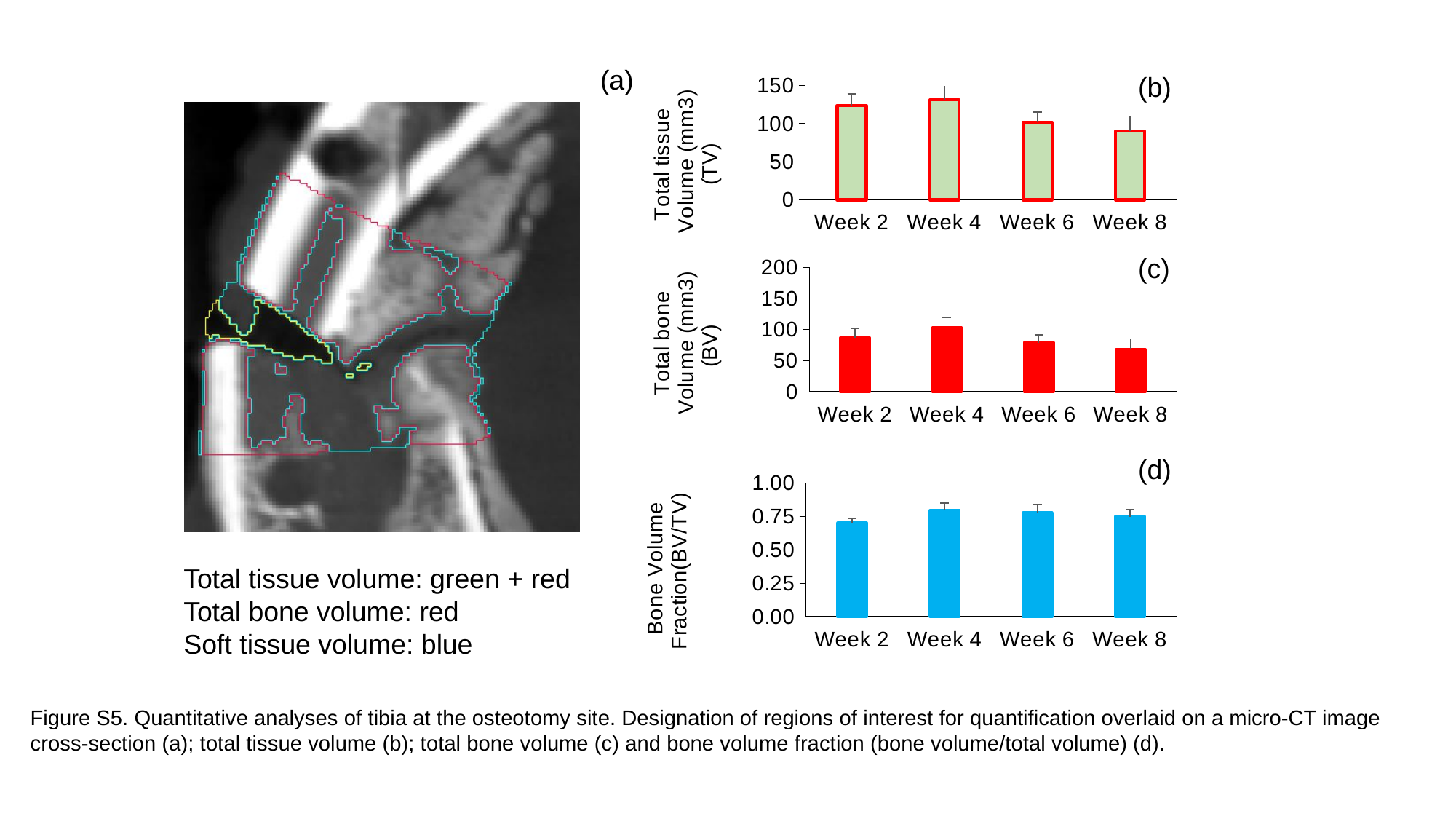

(a)
### Chart
| Category | |
|---|---|
| Week 2 | 123.88965999999999 |
| Week 4 | 131.30318075 |
| Week 6 | 101.98064725 |
| Week 8 | 90.41997775 |(b)
### Chart
| Category | |
|---|---|
| Week 2 | 87.0282975 |
| Week 4 | 103.5715275 |
| Week 6 | 79.14887049999999 |
| Week 8 | 68.1809225 |(c)
(d)
### Chart
| Category | |
|---|---|
| Week 2 | 0.7003816871795607 |
| Week 4 | 0.7968236665406796 |
| Week 6 | 0.7759402971516536 |
| Week 8 | 0.7490876779980746 |Total tissue volume: green + red
Total bone volume: red
Soft tissue volume: blue
Figure S5. Quantitative analyses of tibia at the osteotomy site. Designation of regions of interest for quantification overlaid on a micro-CT image cross-section (a); total tissue volume (b); total bone volume (c) and bone volume fraction (bone volume/total volume) (d).

## Slide 6
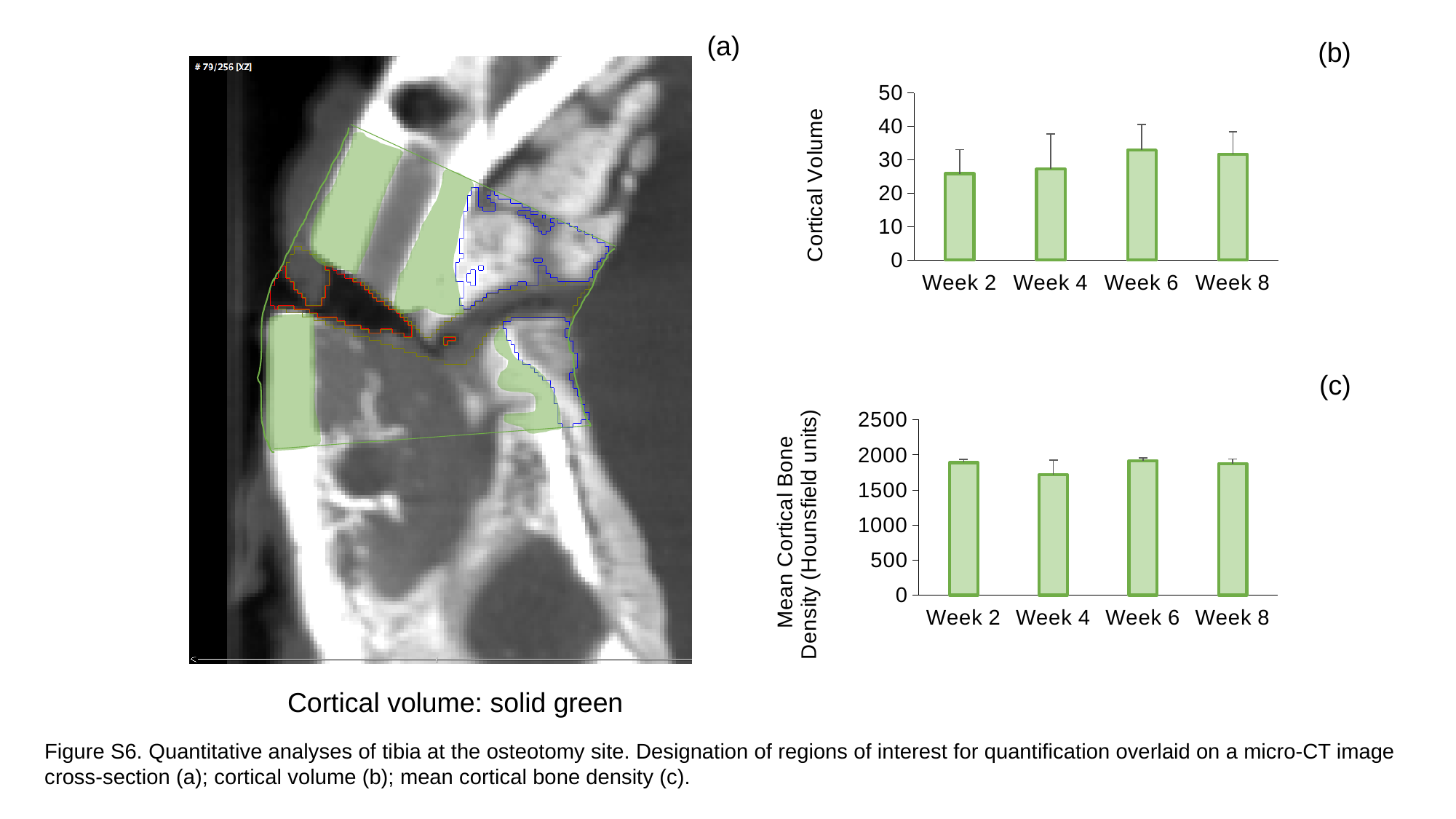

(a)
(b)
### Chart
| Category | |
|---|---|
| Week 2 | 25.8438855 |
| Week 4 | 27.215952 |
| Week 6 | 32.9156325 |
| Week 8 | 31.684410250000003 |
### Chart
| Category | |
|---|---|
| Week 2 | 1889.4849239999999 |
| Week 4 | 1716.76300075 |
| Week 6 | 1917.1350402500002 |
| Week 8 | 1876.78805525 |(c)
Cortical volume: solid green
Figure S6. Quantitative analyses of tibia at the osteotomy site. Designation of regions of interest for quantification overlaid on a micro-CT image cross-section (a); cortical volume (b); mean cortical bone density (c).

## Slide 7
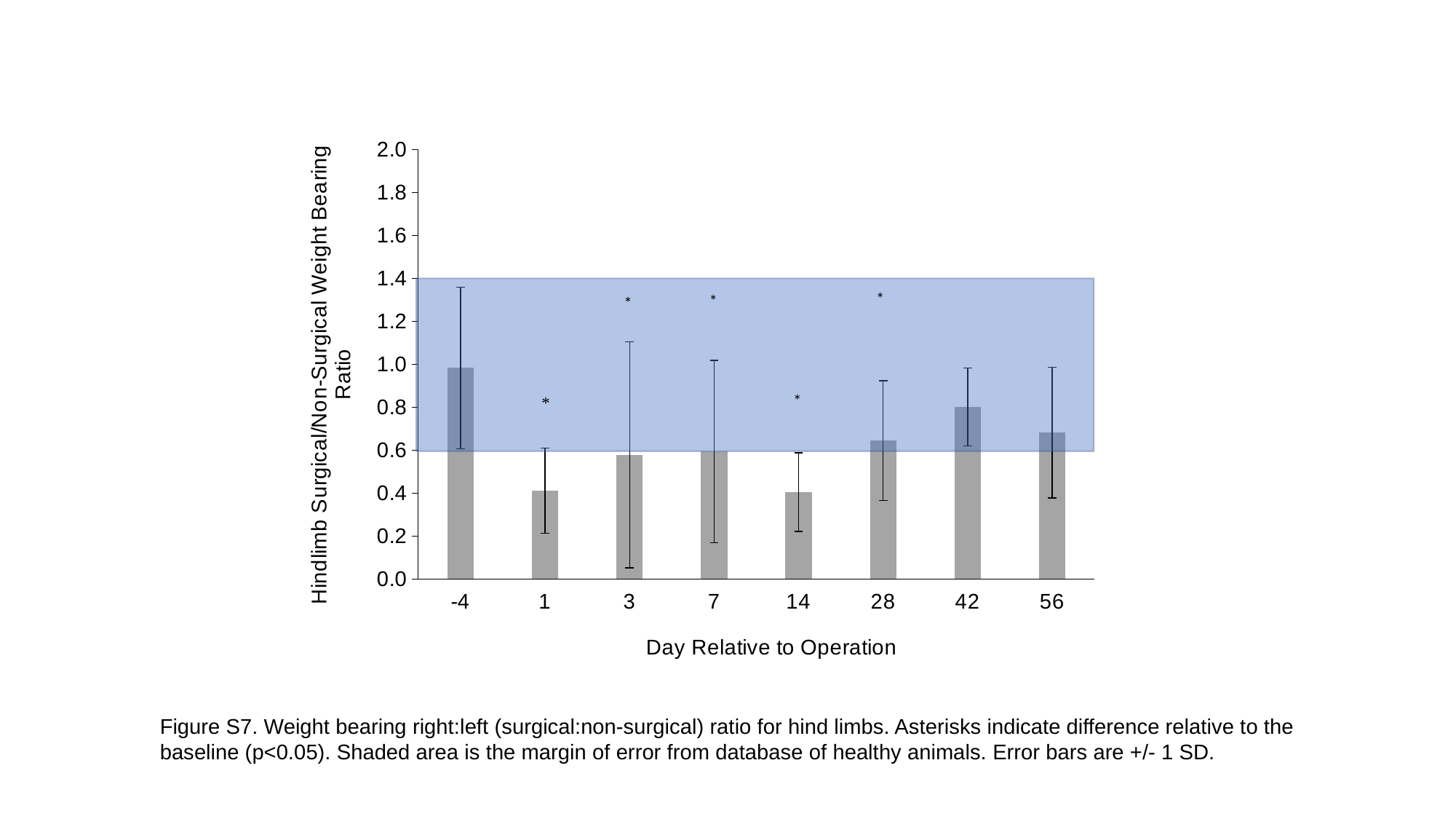

### Chart
| Category | |
|---|---|
| -4 | 0.9826673190650436 |
| 1 | 0.41142572833672836 |
| 3 | 0.5780998021118543 |
| 7 | 0.5937256638918055 |
| 14 | 0.40381125120108385 |
| 28 | 0.6450878670976262 |
| 42 | 0.8010883791606034 |
| 56 | 0.6825249286895285 |Figure S7. Weight bearing right:left (surgical:non-surgical) ratio for hind limbs. Asterisks indicate difference relative to the baseline (p<0.05). Shaded area is the margin of error from database of healthy animals. Error bars are +/- 1 SD.

## Slide 8
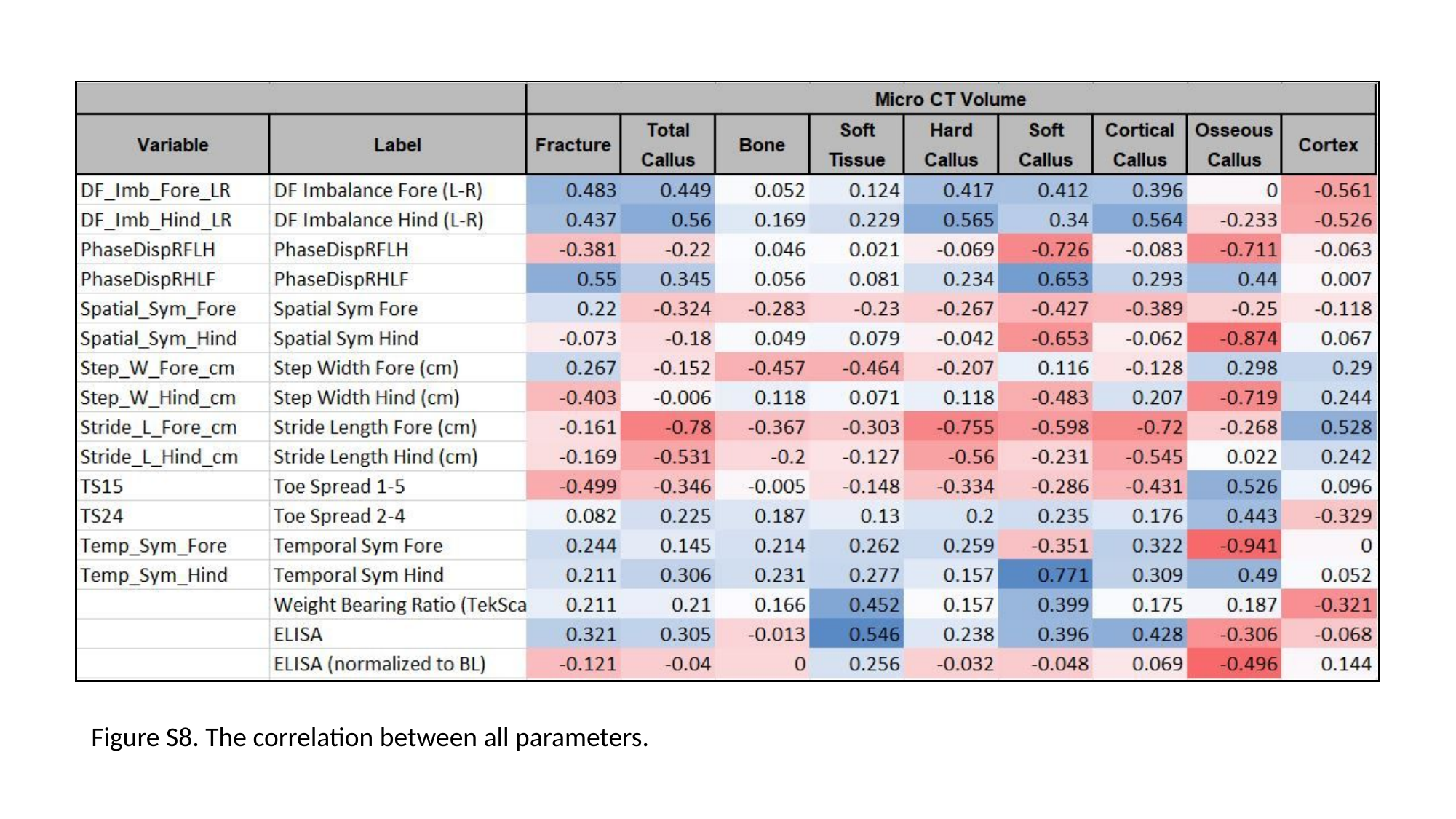

Figure S8. The correlation between all parameters.
