# Supplementary material for: A longitudinal rat model for assessing postoperative recovery and bone healing following tibial osteotomy and plate fixation
Source: BMC Musculoskelet Disord. 2023 Oct 31;24:854. doi: 10.1186/s12891-023-06942-5 (PMC10617055; doi:10.1186/s12891-023-06942-5)
Supplement: Supplementary file 2 — Additional file 2. [file 12891_2023_6942_MOESM2_ESM.docx]

| Table S1. Number of measurements (statistical basis) for α-2-macroglobulin concentration. | | | | | | | | |
| --- | --- | --- | --- | --- | --- | --- | --- | --- |
|  | Pre-operative | Post-operative | | | | | | |
|  |  | Day 1 | Day 3 | Day 7 | Day 14 | Day 28 | Day 42 | Day 56 |
| Number of observations | 16 | 4 | 4 | 4 | 4 | 3 | NA | 4 |
| Number of animals | 17 | 17 | 16 | 16 | 16 | 13 | NA | 5 |

| Table S2. Number of measurements (statistical basis) for weight bearing ratio measurements. | | | | | | | | |
| --- | --- | --- | --- | --- | --- | --- | --- | --- |
|  | Pre-operative | Post-operative | | | | | | |
|  |  | Day 1 | Day 3 | Day 7 | Day 14 | Day 28 | Day 42 | Day 56 |
| Number of observations | 16 | 10 | 13 | 13 | 11 | 10 | 6 | 4 |
| Number of animals | 17 | 17 | 16 | 16 | 16 | 13 | 9 | 5 |

| Table S3a. Number of measurements (statistical basis) for gait parameters (Duty factor imbalance, Temporal Symmetry, Phase Dispersion, Step Width, Step Length, Spatial Symmetry). All measurements are made separately for the fore and hindlimbs except phase dispersion. Two metrics, phase dispersion of right hind-left fore and left hind-right fore are calculated for phase dispersion metrics. | | | | | | | | |
| --- | --- | --- | --- | --- | --- | --- | --- | --- |
|  | Pre-operative | Post-operative | | | | | | |
|  |  | Day 1 | Day 3 | Day 7 | Day 14 | Day 28 | Day 42 | Day 56 |
| Number of observations | 12 | 6 | 8 | 12 | 11 | 9 | 7 | 4 |
| Number of animals | 17 | 17 | 16 | 16 | 16 | 13 | 9 | 5 |

**Table S3b.** The numbers of rats that walked and did not walk on each gait measurement day.

| Table S4. Number of measurements (statistical basis) for 1-5 toe spread measurements. | | | | | | | | |
| --- | --- | --- | --- | --- | --- | --- | --- | --- |
|  | Pre-operative | Post-operative | | | | | | |
|  |  | Day 1 | Day 3 | Day 7 | Day 14 | Day 28 | Day 42 | Day 56 |
| Number of observations | 17 | 16 | 17 | 16 | 16 | 13 | 9 | 5 |
| Number of animals | 17 | 17 | 17 | 16 | 16 | 13 | 9 | 5 |

| Table S5. Number of measurements (statistical basis) for bone healing parameters (micro-CT). | | | | | | | | |
| --- | --- | --- | --- | --- | --- | --- | --- | --- |
|  | Pre-operative | Post-operative | | | | | | |
|  |  | Day 1 | Day 3 | Day 7 | Day 14 | Day 28 | Day 42 | Day 56 |
| Number of observations | NA | NA | NA | NA | 3 | 4 | 4 | 4 |
| Number of animals | 17 | NA | NA | NA | 3 | 4 | 4 | 5 |

| Table S6. Number of measurements (statistical basis) for correlations | | | | | | | | |
| --- | --- | --- | --- | --- | --- | --- | --- | --- |
|  | Pre-operative | Post-operative | | | | | | |
|  |  | Day 1 | Day 3 | Day 7 | Day 14 | Day 28 | Day 42 | Day 56 |
| Hard Callus vs Stride Length, Fore | NA | NA | NA | NA | 2 | 2 | 3 | 0 |
| Soft Callus vs Spatial Symmetry, Hind | NA | NA | NA | NA | 2 | 2 | 3 | 0 |
| Soft Callus vs PD RFLH | NA | NA | NA | NA | 2 | 2 | 3 | 0 |
| Soft Callus vs Temporal Symmetry, Hind | NA | NA | NA | NA | 2 | 2 | 3 | 0 |
| DF Imbalance Hind vs Toe Spread 1-5 | 11 | 5 | 9 | 7 | 14 | 8 | 7 | 3 |
| Stride Length Fore vs Stride Length Hind | 11 | 6 | 9 | 8 | 11 | 8 | 7 | 3 |
| Temporal Symmetry Hind vs Duty Factor Imbalance Hind | 11 | 6 | 9 | 8 | 11 | 8 | 7 | 3 |
| Temporal Symmetry Hind vs Phase Dispersion RHLF | 11 | 6 | 9 | 8 | 11 | 8 | 7 | 3 |
| Temporal Symmetry, Hind vs ELISA | 3 | 1 | 2 | 2 | 3 | 2 | 0 | 3 |
| DF Imbalance Hind vs ELISA | 3 | 1 | 2 | 2 | 3 | 2 | 0 | 3 |
